# Epidemiological Distribution and Subtype Analysis of Premenstrual Dysphoric Disorder Syndromes and Symptoms Based on TCM Theories

**DOI:** 10.1155/2017/4595016

**Published:** 2017-06-15

**Authors:** Mingqi Qiao, Peng Sun, Haijun Wang, Yang Wang, Xianghong Zhan, Hongqi Liu, Xiaoyun Wang, Xia Li, Xiaoru Wang, Jibiao Wu, Fushun Wang

**Affiliations:** ^1^Key Laboratory of Traditional Chinese Medicine for Classical Theory, Ministry of Education, Shandong University of Traditional Chinese Medicine, Jinan, Shandong Province, China; ^2^National Key Subject of TCM Psychology, State Administration of Traditional Chinese Medicine, Jinan, Shandong Province, China; ^3^Laboratory of Ethnopharmacology, Institute of Integrated Traditional Chinese and Western Medicine, Xiangya Hospital, Central South University, Changsha, Hunan Province, China; ^4^School of Preclinical Medical Sciences, Henan University of Traditional Chinese Medicine, Zhengzhou, Henan Province, China; ^5^The Affiliated Hospital of Shanxi University of Traditional Chinese Medicine, Taiyuan, Shanxi Province, China; ^6^The Second Affiliated Hospital of Guangzhou University of Chinese Medicine, Guangzhou, Guangdong Province, China; ^7^The Central Hospital of Jinan City, Jinan, Shandong Province, China; ^8^Department of Psychology, Nanjing University of Chinese Medicine, Nanjing, Jiangsu Province, China

## Abstract

We performed an epidemiological investigation of subjects with premenstrual dysphoric disorder (PMDD) to identify the clinical distribution of the major syndromes and symptoms. The pathogenesis of PMDD mainly involves the dysfunction of liver conveyance and dispersion. Excessive liver conveyance and dispersion are associated with liver-qi invasion syndrome, while insufficient liver conveyance and dispersion are expressed as liver-qi depression syndrome. Additionally, a nonconditional logistic regression was performed to analyze the symptomatic features of liver-qi invasion and liver-qi depression. As a result of this analysis, two subtypes of PMDD are proposed, namely, excessive liver conveyance and dispersion (liver-qi invasion syndrome) and insufficient liver conveyance and dispersion (liver-qi depression syndrome). Our findings provide an epidemiological foundation for the clinical diagnosis and treatment of PMDD based on the identification of different types.

## 1. Introduction

With a prevalence rate of 3%–8% [1–3], premenstrual dysphoric disorder (PMDD) is characterized by a group of symptoms that manifest as emotional disorders and painful breast distension. PMDD can significantly affect one's work and social functioning one week before the menstrual period. Different subtypes of PMDD have been described [4–8], and selecting the appropriate treatment for the subtype is an important consideration for management. As early as 1983, it was suggested that premenstrual syndrome (PMS) could be divided into four subtypes, namely, PMS-A (anxiety), PMS-D (depression), PMS-C (cravings), and PMS-H (hyperhydration) [9]. Subsequently, a large number of clinical studies demonstrate that fluoxetine, the first-line treatment for PMDD, can address some of the symptoms of PMDD, including dysphoria [10], food cravings [11], tension, and anxiety [12–14], but it has no effect on other clinical manifestations, such as sexual hypoactivity, indicating that PMDD has subtypes that differ in their pathogenesis. The effective rate of fluoxetine treatment is less than 60% [15], which may be due to the existence of different central mechanisms for the different PMDD subtypes. Through epidemiological investigations in China [16] it has been shown that the four syndromes of liver-qi invasion, liver-qi depression, liver fire flaring, and heart-spleen deficiency account for 95% of the symptoms of PMDD (58.9% and 27.5% for liver-qi invasion and liver-qi depression, resp.), and appropriate treatments, such as Jingqianping granules and Jingqianshu granules, have been developed.

Based on the findings described above, the existence of PMDD subtypes has been recognized by the medical community both in China and abroad, but the classification systems and treatment goals vary. This research was conducted as an epidemiological investigation of the relationship between TCM syndromes and the symptoms of PMDD. Subtypes of PMDD are described based on the pathogenic mechanisms addressed in TCM and on the symptoms reported by the patients.

## 2. Materials and Methods

### 2.1. Ethics Statement

This study was approved by Shandong University of Traditional Chinese Medicine and the State Administration of Traditional Chinese Medicine. The patients and healthy volunteers who participated in this study all signed informed consent forms.

### 2.2. Inclusion and Exclusion Criteria

Inclusion criteria included symptoms consistent with PMDD as described in the Diagnostic and Statistical Manual of Mental Disorders (DSM-IV); women ranging in age from 18 to 45 years, with no exclusion based on race; possessing clear consciousness and independence as well as active judgment; being able to understand the research goals and willing to cooperate; no severe disease of the heart, liver, or kidney, no brain tumor or other craniocerebral diseases, and no history of taking psychotropic drugs.

The diagnostic criteria for liver-qi invasion and liver-qi depression are based on the results of previous epidemiological research,* “The 7th Edition of Basic Theory of TCM,”* and the Delphi method. The diagnostic criteria for the diagnoses of liver depression forming fire, liver stagnation and spleen deficiency, liver depression and blood stasis, liver depression and kidney deficiency, and spleen and kidney deficiency syndromes are based on* “Diagnostics of TCM,*” “*Guiding Principles of Clinical Research on TCM New Drugs,”* and the Delphi method. The diagnostic criteria for liver-qi invasion and liver-qi depression syndromes are described below.

Diagnostic criteria for liver-qi invasion syndrome are as follows: major symptoms: premenstrual dysphoria and irritability; painful distension of the breasts; decreased function in work, household management, and social activity; minor symptoms: distension in the head, headache, chest distress, anxiety, dreaminess, constipation, and premature menstruation; tongue vein: light red or red tongue, white or slightly yellow coating, and a weak pulse. Diagnosis: the diagnosis of liver-qi invasion is confirmed if two of the major symptoms and three or more of the minor symptoms are present in combination with the characteristics of the tongue vein.

Diagnostic criteria for liver-qi depression syndrome are as follows: major symptoms: low spirits or depression and anger during the premenstrual phase; chest distress; decreased function in work, household management, and social activity; minor symptoms: painful distension of the breasts, chest-coerced distending pain, preference for sigh, abdominal bulge, anorexia, loss of strength, dysmenorrhea, reduced discharge during menses, and delayed menstruation; tongue vein: slightly dark or dark tongue, fat tongue body, slightly white or yellow coating, and a weak or slow pulse. Diagnosis: the diagnosis is confirmed if two of the major symptoms (poor mood or depression and anger during the premenstrual phase is required) and three of the minor symptoms are present in combination with the characteristics of the tongue vein.

Exclusion criteria are as follows: psychiatric patients; patients with severe physical diseases; a history of drug abuse (including the use of drugs to treat PMDD within three months); hematological diseases; pregnancy or lactation; patients who are unable to cooperate during visits due to aphasia, a disturbance of consciousness, dementia, or other conditions; patients with chronic five-organ diseases and clinical symptoms; patients who had undergone unilateral oophorectomy or abortion within six months.

### 2.3. Patient Demographics

An investigation of 3,530 cases of child-bearing women from Shandong, Henan, Guangzhou, and Shanxi provinces was conducted. In total, 675 cases were selected for observation, including 107 cases from Shandong Traditional Chinese Medicine University, 250 cases from Henan College of Traditional Chinese Medicine and its affiliated hospital, 149 cases from The Second Affiliated Hospital of Guangzhou University of Traditional Chinese Medicine, and 169 cases from the Affiliated Hospital of Shanxi College of Traditional Chinese Medicine.

Ethnicity: the cases included 662 Han Chinese (98.1%) and 13 individuals of other ethnicities (1.9%); age: 521 subjects were aged 18–28 (77.2%), 118 subjects were aged 29–39 (17.5%), and 31 subjects were aged 40–48 (4.6%); marriage status: 513 subjects were unmarried; 162 were married; profession: 391 subjects were students (57.9%), 91 were office clerks (13.5%), 33 were self-employed (4.9%), 34 were doctors (5.0%), 10 were teachers (1.5%), 14 were civil servants (2.1%), 30 were unemployed (4.5%), 8 were laborers (1.2%), 2 were farmers (0.3%), and the profession was unknown for 62 subjects (9.2%); educational level: 496 subjects had a college education (73.5%), 96 had a postgraduate education (14.2%), 61 had a high school (technical secondary school) education (9.0%), 15 had a middle school education (2.2%), and 7 had a primary school education or below (1.0%).

### 2.4. Methodology

This study was designed as a cross-sectional epidemiological investigation [17–19]. The survey included items related to the participants' general information and medical history, the Information Collection Table of PMDD TCM Symptoms, information for TCM syndrome differentiation (based on expert experience), and a daily record of the severity of problems (DRSP). Frequency analysis and nonconditional logistic regression analysis [20, 21] were performed to evaluate the PMDD subtypes in combination with the pathogenesis and symptoms according to TCM.

## 3. Results

### 3.1. Distribution of PMDD Syndromes (675 Cases)

Through a cross-sectional epidemiological analysis of the 675 patients with PMDD, a frequency analysis was performed to summarize the distribution of PMDD syndromes, as shown in Table 1.

The major syndromes refer to the primary syndromes of the disease. A patient will present a specific syndrome; for example, liver-qi depression is a major syndrome of PMDD. Minor syndromes indicate the presence of the major syndrome of the disease, and the patient may also present another syndrome with a relatively complete manifestation. For example, liver-qi depression syndrome, a major syndrome of PMDD, is accompanied by liver and kidney deficiency syndrome. The accompanying syndromes are expressions or symptoms of other syndromes associated with the disease. The key difference between the accompanying syndromes and the minor syndromes mainly lies in whether a complete manifestation of the syndrome is present.

From Table 1, it can be seen that the major syndromes of PMDD include liver-qi invasion and liver-qi depression. Liver stagnation and spleen deficiency, liver depression forming fire and liver depression, and blood stasis are also common. The syndromes of liver depression and kidney deficiency, as well as spleen and kidney deficiency, were present at relatively low frequencies, but they tended to emerge as nonmajor syndromes. The pathogenesis of most of these syndromes is related to the liver, with the dysfunction of liver conveyance and dispersion playing the most important role.

### 3.2. Distribution of PMDD Symptoms (675 Cases)

Through a cross-sectional epidemiological analysis of the 675 patients with PMDD, a frequency analysis was conducted to summarize the distribution of the PMDD symptoms, as shown in Table 2.

From Table 2, it can be seen that there are three types of PMDD symptoms, namely, emotional symptoms, physical symptoms, and symptoms related to social function. Major symptoms include the following: emotional symptoms (impatience and irritability, emotional depression, anxiety, low spirits, and hopeless feelings) and physical symptoms (fatigue, cold intolerance and cold limbs, insomnia, dreaminess, head distension, headache, dizziness, dry mouth and throat, chest distress, chest-coerced distension, painful distension of the breasts, preference for sighing, anorexia, abdominal bulge, painful distension of the abdomen, soreness and weakness of the waist and knees, loose stool, sexual hypoactivity, dysmenorrhea, reduced discharge during menses and dark purple menstrual flow, and impaired social function affecting the ability to work, manage household tasks, and learn).

The typical clinical manifestation of PMDD is dominated by emotional symptoms, such as impatience and irritability, as well as emotional depression. Impatience and irritability are typical symptoms of excessive liver conveyance and dispersion and emotional depression is associated with insufficient liver conveyance and dispersion. The liver-qi invasion and liver-qi depression syndromes play a dominant role in the PMDD syndromes. Therefore, analyzing the symptomatic features of the liver-qi invasion and liver-qi depression syndromes is important for establishing the PMDD subtype.

### 3.3. Analysis of the Symptomatic Features of PMDD Liver-Qi Invasion Syndrome

With the presence or absence of liver-qi invasion syndrome as the dependent variable and the relevant symptoms as independent variables, a nonconditional logistic regression analysis was performed to analyze the symptomatic features of PMDD liver-qi invasion syndrome, as shown in Table 3.

The results show that impatience and irritability, head distension, headache, painful distension of the breasts, abdominal bulge, and loose stool tended to emerge as a group of internally related symptoms, demonstrating the pathogenic features of qi abnormal rising for excessive liver conveyance and dispersion.

### 3.4. Analysis of the Symptomatic Features of PMDD Liver-Qi Depression Syndrome

With the presence or absence of liver-qi depression as the dependent variable and the relevant symptoms as the independent variables, a nonconditional logistic regression analysis was performed to analyze the symptomatic features of PMDD liver-qi depression syndrome, as shown in Table 4.

The results show that emotional depression, painful distension of the breasts, and reduced discharge during menses represent a group of internally related symptoms, demonstrating the pathogenic features of qi stagnation due to insufficient liver-qi conveyance and dispersion.

## 4. Discussion

To improve readers' ability to understand this research, efforts have been made to use English terminology to discuss the concepts and words used in traditional Chinese medicine (TCM). Although some of the concepts in TCM have no corresponding words in English and there is no standard reference for the translation of certain expressions, we hope that our interpretations (Table 5) will aid in the understanding of the theories that underlie TCM. The concepts used in TCM have been shown to be effective and can guide treatment decisions in the clinic; nonetheless, TCM remains marginalized in current medical practice. The explanations of relevant concepts and expressions do not comprehensively describe TCM theory, but they are intended to provide readers with a better understanding of the research content and original results described in this paper.

Based on the studies described above, the pathogenic mechanism of PMDD is mainly related to the disorder of liver conveyance and dispersion, thus involving the spleen and kidneys. The pathogenic site mainly involves the circulating parts of the jueyin liver meridian of the foot; therefore, differentiation is based on the liver. The liver controls conveyance and dispersion, and liver dysfunction can cause excessive or insufficient conveyance and dispersion. Symptoms such as impatience and irritability accompanied by head distension, headache, dizziness, painful distension of the breasts, abdominal bloating, loose stool, and others are clinical manifestation of excessive conveyance and dispersion. Insufficient conveyance and dispersion are characterized by emotional depression and low spirits accompanied by painful distension of the breasts and reduced discharge during menses. Apart from the occurrence of painful distension of the breasts, which occurs in both excessive and insufficient conveyance and dispersion, the symptoms listed above can differentiate the pathogenesis of PMDD, demonstrating that there are at least two relatively independent clinical subtypes, namely, liver-qi invasion and liver-qi depression syndromes. Because other syndromes, such as liver depression forming fire, liver stagnation and spleen deficiency, liver depression and blood stasis, and liver depression and kidney deficiency, are components of liver-qi invasion and liver-qi depression syndromes and are less distributed among patients; they lack discriminatory power. This research confirms that there are at least two primary subtypes of PMDD, namely, liver-qi invasion and liver-qi depression syndromes.

Liver-qi invasion syndrome indicates excessive qi conveyance and dispersion as well as qi and blood rising; therefore, the jueyin liver meridian of the foot prompts impatience and irritability, head distension, headache, dizziness, and chest distress. Liver-qi depression indicates insufficient qi conveyance and dispersion; therefore, emotional depression, low spirits, no liver upbearing and effusion, unsmooth qi flow, and reduced discharge during menses are reported. Painful distension of the breasts is a common manifestation of disturbances of qi movement in the liver meridian, demonstrating that the two subtypes (syndromes) have a common mechanism but different features. Compared with the subtype classifications used throughout the world, the PMDD subtypes described in this paper are characterized by obvious features that are differentiated according to TCM and provide valuable clinical guidance.

## 5. Conclusion

Liver dysfunction is the major pathogenic feature used in the differentiation of the clinical syndromes of PMDD. Excessive and insufficient liver conveyance and dispersion can cause two types of clinical manifestations, pointing to the existence of independent subtypes of PMDD. Suppressing a hyperactive liver to address descending adverse qi and discharging the liver to relieve depression can provide improved clinical results.

## Figures and Tables

**Table 1:** Distribution of syndromes in 675 PMDD patients (case number, occurrence frequency).

Syndrome	Liver-qi invasion	Liver-qi depression	Liver depression forming fire	Liver stagnation and spleen deficiency	Liver depression and blood stasis	Liver depression and kidney deficiency	Spleen and kidney deficiency
Major	220 (32.6%)	234 (34.7%)	62 (9.2%)	70 (10.4%)	53 (7.9%)	25 (3.7%)	13 (1.9%)
Minor	32 (4.7%)	56 (8.3%)	43 (6.4%)	74 (11.0%)	50 (7.4%)	45 (6.7%)	57 (8.4%)
Both (accompanying)	16 (2.4%)	33 (4.9%)	79 (11.7%)	57 (8.4%)	99 (14.7%)	76 (11.3%)	47 (7.0%)
None	407 (60.3%)	352 (52.1%)	491 (72.7%)	474 (70.2%)	473 (70.1%)	529 (78.4%)	558 (82.7%)

**Table 2:** Distribution of symptoms in 675 PMDD patients (case number, occurrence frequency).

Emotional symptoms	Impatience & irritability 600 (88.9%)	Emotional depression 582 (86.2%)	Anxiety 490 (72.6%)	Low spirits 543 (80.4%)	Hopeless feelings 307 (45.5%)	
Social functions	Decreased work ability 578 (85.9%)	Decreased household management ability 532 (79.1%)	Decreased learning ability 575 (85.1%)			

Physical symptoms	Fatigue 612 (90.7%)	Edema 139 (20.5%)	Cold intolerance & cold limbs 361 (53.5%)	Insomnia 231 (32.8%)	Dreaminess 438 (64.2%)	Head distension 234 (34.6%)
	Headache 233 (34.5%)	Dizziness 266 (39.0%)	Dry mouth and throat 406 (60%)	Chest distress 323 (47.5%)	Chest-coerced distension 278 (40.6%)	Painful distension of the breasts 495 (73.3%)
	Preference for sighing 457 (67.7%)	Anorexia 291 (42.7%)	Abdominal bulge 532 (78.8%)	Painful distension of the abdomen 454 (67.2%)	Waist and knee soreness and weakness 491 (72.7%)	Loose stool 268 (39.6%)
	Constipation 174 (25.7%)	Frequent urination 153 (22.7%)	Sexual hypoactivity 372 (55.1%)	Dysmenorrhea 493 (73.5%)	Reduced discharge during menses 368 (54.8%)	Dark purple menstrual flow 420 (62.6%)

**Table 3:** Results of a nonconditional logistic regression analysis of the symptomatic features of liver-qi invasion syndrome.

	*B*	SE	Wald	Sig.	Exp (*B*)
Impatience & irritability	0.985	0.141	49.005	0.000	2.677
Fatigue	−0.058	0.137	0.181	0.670	0.943
Head distension	0.135	0.172	0.615	0.433	1.144
Headache	0.075	0.141	0.278	0.598	1.077
Dizziness	0.408	0.150	7.436	0.006	1.504
Painful distension of the breasts	0.054	0.122	0.199	0.656	1.056
Chest-coerced distension	−0.028	0.189	0.023	0.881	0.972
Chest distress	0.095	0.126	0.575	0.448	1.100
Preference for sighing	−0.254	1.120	4.481	0.034	0.775
Abdominal bulge	0.030	0.125	0.059	0.808	1.031
Waist and knee soreness and weakness	−0.214	0.111	3.728	0.053	0.807
Loose stool	0.021	0.119	0.030	0.862	1.021
Constant	−3.005	0.489	37.757	0.000	0.050

**Table 4:** Results of a nonconditional logistic regression analysis of the symptomatic features of liver-qi depression syndrome.

	*B*	SE	Wald	Sig.	Exp (*B*)
Emotional depression	0.476	0.117	16.427	0.000	1.610
Fatigue	−0.166	0.129	1.647	0.199	0.847
Chest distress	−0.116	0.120	0.949	0.330	0.890
Chest-coerced distension	−0.030	0.178	0.028	0.868	0.971
Painful distension of the breasts	0.076	0.118	0.415	0.519	1.079
Preference for sighing	−0.114	0.111	1.041	0.308	0.893
Anorexia	−0.096	0.112	0.728	0.394	0.909
Abdominal bulge	−0.103	0.118	0.772	0.380	0.902
Waist and knees soreness and weakness	−0.176	0.103	2.882	0.090	0.839
Dysmenorrhea	−0.023	0.192	0.014	0.906	0.978
Reduced discharge during menses	0.134	0.180	0.551	0.458	1.143
Dark purple menstrual flow	−0.162	0.177	0.837	0.360	0.851
Sexual hypoactivity	−0.071	0.106	0.447	0.504	0.931
Constant	0.427	0.550	0.603	0.437	1.532

**Table 5:** Concepts and words used in traditional Chinese medicine (TCM).

Concepts or words used in TCM	Interpretation
Theory of TCM	A general term for the medical theories of the Chinese people, which have been used in practice and to guide treatment decisions since before the introduction of Western medicine into China. The main difference between TCM and Western medicine can be summarized as follows: Western medicine stems from Greek philosophy and advocates intervening in the focal disease using the method of logic. TCM originates from Chinese classical philosophy and advocates interventions aimed at the whole human and the living environment using the method of delogic.

TCM	TCM mainly refers to nonsynthetic preparations, such as natural extractives and herbal extracts. Researchers in the field of TCM adopt different attitudes. Some clinicians maintain that TCM should be replaced with a pharmacological approach; in other words TCM theory should be abandoned, and the pharmacological approach of modern medicine should be used to determine the effectiveness of drugs and to differentiate between a large number of therapeutic options quickly and effectively, with the aim of retaining effective TCMs and discarding useless drugs. Other doctors who practice TCM believe that TCM should not be submitted to the logic of modern medicine to evaluate its effectiveness. The reason that TCM is defined as medicine according to TCM theory lies in Chinese classical philosophy, in which a nonlogic or delogic view is applied. According to this philosophy, the human body or the environment is taken as a whole when considering the intervention. This research does not involve TCM intervention; therefore, this paper does not express an opinion on TCM.

Qi	TCM philosophy holds that qi is a type of constantly moving and extremely impalpable material within the human body as well as a basic material that constitutes and maintains life.

Syndrome	A generalization of the pathological attributes that are present at a certain stage in the development of a disease.

Liver-qi	Liver dysfunction, qi movement stagnation, and the pathological changes of liver-qi disruption, invading the spleen and stomach.

Liver-qi stagnation	The liver has the function of conveyance and dispersion. For example, dysfunction of liver conveyance and dispersion or emotional depression can cause liver-qi stagnation. Clinical manifestations include hypochondriac pain, chest distress, epigastric distension, belching, and irregular menstruation.

Liver fire flaming	This term refers to a flaming fire that disturbs the liver and causes liver-qi invasion. It is associated with heat-related syndromes, including headache, irritability, ear ringing, hypochondriac pain, and other heat syndromes.

Heart and spleen deficiency	This term refers to a syndrome that is common in heart blood insufficiency and spleen-qi deficiency. The main manifestations include palpitation, dreaminess, forgetfulness, reduced appetite, abdominal distension, sloppy stool, lack of strength, uterine bleeding, hematochezia, subcutaneous hemorrhage, pale tongue, and weak pulse.

Liver depression forming fire	This term refers to a type of TCM syndrome involving chest hypochondrium, abdominal distension and pain, impatience and irritability, dizziness and swelling pain, flushed face and congested eyes, bitter taste, dry throat, sleeplessness, painful distension of the breasts, irregular menstruation, and even amenorrhea due to liver dysfunction of conveyance and dispersion as well as stagnation of qi movement caused by mental dissatisfaction, emotional provocation, and disease invasion.

Liver stagnation and spleen deficiency	The syndromes of liver and spleen dysfunction, dominated by chest-coerced distending pain, abdominal distension, and loose stool.

liver depression and blood stasis	The common syndromes include coerced distending pain or sharp pain, a lump under the costal region or at the abdomen, emotional depression, dark purple tongue or spots on the tongue, and an irregular pulse caused by liver-qi stagnation and blood stasis in liver.

Liver depression and kidney deficiency	The liver and kidney influence each other physiologically and are pathologically related due to their shared sources. Generally, liver depression and kidney deficiency are dominated by a deficiency of kidney-yang and caused by the lack of warming and nourishing of the liver-yang. Deficiency of kidney-yin is caused by a lack of nourishing of the liver-yin, by kidney-yin and liver-yin not controlling liver-yang, and by failure of the kidneys to nourish the liver. The spread of liver disease to the kidney is caused by mental dissatisfaction, depression transformed to fire, and prolonged disease affecting kidney. Therefore, the liver and kidneys influence each other pathologically.

Spleen and kidney deficiency	This syndrome tends to occur in diseases such as consumptive disease, dysentery, edema, tympanites, chronic gastroenteritis, chronic nephritis, and chronic renal failure.
